# Internet-based digital intervention to support the self-management of hypertension compared to usual care: results of the HALCYON randomized controlled trial

**DOI:** 10.1186/s12872-025-04698-5

**Published:** 2025-04-04

**Authors:** Björn Meyer, Antje Riepenhausen, Linda T. Betz, Kamila Jauch-Chara, Alexander Reshetnik

**Affiliations:** 1 GAIA, Hamburg, Germany; 2 Medical Faculty, Christian-Albrechts-Universität zu Kiel, Kiel, Germany; 3 Department of Nephrology and Intensive Care Medicine, Charité, Freie Universität Berlin and Humboldt-Universität zu Berlin, Berlin, Germany

**Keywords:** Behavioral change, Cognitive behavioral therapy, Digital intervention, Hypertension, RCT

## Abstract

**Background:**

Hypertension is a major public health problem. Despite existing treatment options, overall blood pressure control is still insufficient. Digital health interventions have the potential to improve treatment success. We developed *liebria*, an internet-based digital intervention accessible via smartphones and computers, designed to support the self-management of hypertension.

**Methods:**

We tested the effectiveness of *liebria* in reducing systolic blood pressure and improving other relevant endpoints in adults with hypertension using a parallel randomized controlled trial design. Adults with hypertension (mean age 54.5 years, 47.1% male) were recruited via a German health insurance provider and randomized (1:1; concealed allocation; no blinding of participants) to receive *liebria* in addition to treatment as usual (*n* = 52), or treatment as usual alone (*n*=50). Primary outcome was systolic blood pressure after 3 months (T1). Secondary endpoints were diastolic blood pressure, pulse pressure, quality of life, medication adherence, and social and work-related functioning.

**Results:**

The study's statistical power was limited due to a smaller sample size (*N* = 102) than the a priori target sample size (*N*=676). Intention-to-treat analyses showed an effect of *liebria* on systolic blood pressure (baseline-adjusted between-group difference -3.5 mmHg, 95% CI -7.13 to 0.13, *p* = .053, Cohen’s *d* = 0.36). More participants in the intervention group (78.2% vs. 54.3% in the control group) showed reductions in systolic blood pressure (*p* = .076). Moreover, *liebria* had effects on social and work-related functioning. No effects emerged for diastolic blood pressure, pulse pressure, quality of life, or medication adherence. No adverse events or device effects were observed.

**Conclusions:**

The present study shows first promising results of *liebria*’s effects on systolic blood pressure and social and work-related functioning. Future studies should aim to replicate effects in a larger sample to increase statistical power.

**Trial registration:**

German Clinical Trials Register (DRKS00025871); https://drks.de/search/en/trial/DRKS00025871 ; date of registration: October 5, 2021.

**Supplementary Information:**

The online version contains supplementary material available at 10.1186/s12872-025-04698-5.

## Background

Worldwide, an estimated 40% of adults are diagnosed with, and around ten million deaths per year can be attributed to arterial hypertension, making it the leading risk factor for disability-adjusted life years (DALYs) and premature death [1, 2]. Lowering elevated blood pressure levels is associated with clinically significant reductions in the risk for cardiovascular disease (CVD) and death [3–6], and is therefore of utmost importance for reducing overall mortality.

Therapeutic procedure usually consists of non-pharmacological strategies targeting weight loss, diet, physical activity, and reducing alcohol consumption alone or combined with antihypertensive medical treatment [7]. However, many patients fail to achieve satisfactory blood pressure control despite the availability of a range of effective hypertension management strategies [8, 9]. On the one hand, general practitioners have limited time for the discussion of hypertension management with their patients [10, 11]. In busy clinical practice, physicians are not always able to give detailed and accurate advice on specific non-pharmacological strategies. In addition, compliance problems are well known in the treatment of hypertension [12–14]. Thus, there is a need for assistance in integrating health-promoting behaviors into the lives of patients with hypertension to prevent complications such as hypertensive crises and long-term cardiovascular sequelae [15].

Digital therapeutics (DTx) can address these challenges in hypertension control by conveying relevant educational content, improving patients’ motivation to maintain healthy behaviors and to adhere to medication schedules, and applying relevant behavior-change techniques (e.g., goal-setting, self-monitoring) [16–18]. Such interventions could be integrated into existing treatment structures as an adjunctive treatment to current standard of care, supporting physicians and patients in hypertension management. Access to these DTx is flexible and convenient, making them ideal for supporting non-pharmacological treatment options for hypertension in everyday life [19, 20]. To date, several DTx have been shown to be effective in improving medication adherence in hypertensive patients [21, 22] as well as BP control [20, 23, 24]. The majority of these interventions focus on individual, or a combination of several, management elements such as (self-)monitoring [25–31], reminders to increase medication adherence [21, 22, 27, 30, 32–34] or education about healthy lifestyle [27, 30, 34–36]. Some interventions also include behavior change coaching [35, 37], but to our knowledge, none of the currently available interventions is completely scalable (i.e., merely relying on digital content) while also regularly assessing and incorporating patient feedback. Such an interactive intervention design would enable an eminent possibility of tailoring the content to the patient while keeping costs low.

The aim of the HALCYON trial (Effectiveness of a **H**ypertension-focused Digital **A**pp**L**ication in a **C**ommunit**Y** Sample: Randomized C**ON**trolled Trial) was to investigate the effectiveness of the DTx *liebria* that uses the form of an interactive simulated dialogue to address a variety of topics relevant to BP management including education, behavior change techniques, self-monitoring, and parasympathetic training in reducing systolic blood pressure (SBP) in adults with hypertension. This DTx followed the same design and development approach used by our team for similar evidence-based interventions addressing conditions such as depression, anxiety disorders, substance use disorders, breast cancer survivorship, epilepsy, and multiple sclerosis [38–44]. The trial investigated *liebria*’s effect on different endpoints related to blood pressure, quality of life, medication adherence, and functioning. This pragmatic trial aimed to assess how adding *liebria* to heterogeneous routine care in Germany would impact these endpoints over three months; therefore, the control group continued with their usual care, reflecting real-world clinical practice.

## Methods

### Recruitment and assessment

This study was approved by the ethics committee of the medical faculty at the Christian-Albrechts-Universität zu Kiel (D 560/21) and registered in the German Clinical Trials Register (https://drks.de/search/en/trial/DRKS00025871). Recruitment was conducted via a disease-specific campaign orchestrated by a health insurance company (DAK-Gesundheit) in Germany. Interested participants were invited to visit a study website that provided information about the study and could indicate their interest in participating using a contact form. After participants provided electronic informed consent, eligibility for the study was assessed via a screening process that included questionnaires and home-based blood pressure measurements (HBPM), self-reported by the participants. When inclusion criteria were met, participants were randomized to one of two study groups (intervention or control group) using an automated randomization sequence generation software, with an allocation ratio of 1:1 (no blocked randomization, no stratification, concealed allocation, no blinding of participants). For the duration of the trial, the intervention group (IG) received access to *liebria* in addition to treatment as usual (TAU), while participants randomized to the control group (CG) only had access to TAU. Participants of both groups were recontacted after three months (T1) to again complete online assessments and HBPM. All procedures followed were in accordance with institutional guidelines. All study procedures took place online.

### Inclusion and exclusion criteria

The following inclusion criteria applied:

- age ≥ 18;
- hypertension (≥ 135/85 mmHg and < 175/105 mmHg in home-based monitoring);
- no initial prescription or change in existing antihypertensive drug therapy in six weeks prior to study enrolment;
- sufficient understanding of the German language;
- consent to participation.

The following exclusion criteria applied:

- current prescription of more than three antihypertensive agents;
- secondary cause of arterial hypertension (i.e. hypertension caused by thyroid disease, kidney disease or other diseases);
- pregnancy.

Of note, patients prescribed more than three antihypertensive agents were excluded to focus on a population with greater behavioral treatment potential. Those with treatment-resistant hypertension typically require specialized pharmacological management, which could introduce high variability in study outcomes.

### Intervention

The intervention *liebria* is a DTx for adults with primary hypertension that can be accessed via the Internet on users’ personal devices, such as desktop computers, laptops, or smartphones. *liebria* uses methods from cognitive behavioral therapy (CBT) and lifestyle change counseling, covering topics such as clarifying and strengthening motivation, training the parasympathetic nervous system, recognizing and overcoming obstacles, or strengthening impulse control (for details, see Table 1**;**for a visual impression, see Figure S1). The program is presented as a simulated dialog that adapts to the user's preferences and needs, where the user interacts with the program by selecting one or more predefined response options. A number of additional features are offered, including audio recordings to guide therapeutic exercises, downloadable PDF materials, customized motivational messages delivered via SMS or email, and self-monitoring questionnaires to track target behaviors. Participants were encouraged to use *liebria* flexibly, according to their personal schedule and preferences. The program provides guidance on available topics and estimated session duration, with a general recommendation to engage approximately two to three times per week for about 15 minutes per session.

**Table 1:** Detailed description of the intervention *liebria
*

**Module**	**Description**
**Introduction**	Interactive exploration of current symptoms (e.g. severity of hypertension, physical symptoms); education on the goals and functions of the program; education on the etiology of hypertension and the role of health-promoting behavioral habits; exploration of personal goals and creation of an individual “health goal profile” (e.g. losing weight, reducing stress, healthier eating habits, low-salt diet, smoking / reducing alcohol consumption); introduction to the technique of "decelerated breathing" to reduce blood pressure; mental imagery audio exercise (visualization of the "healthy future self"); homework: Repeat audio exercise and work on personal health goals; symbolic reward for progress (“puzzle piece of the day”).
**Clarifying and strengthening motivation**	Discussion of homework and personal goal progress; interactive exploration of the topic of change motivation with a fictitious case example; interactive exercise to clarify and strengthen personal motivation and clarify core values (“Why do I really want to lower my blood pressure?”); homework: regular breathing exercises, pursue health goals, implement personal values in everyday life; audio exercise on motivation and personal values; symbolic reward for progress (“puzzle piece of the day”).
**Training the parasympathetic nervous system**	Discussion of personal goal progress; theoretical overview of the topic of parasympathetic training to reduce hypertension ("how slow breathing could lower your blood pressure"; role of the vagus nerve, techniques for vagus nerve stimulation such as cold stimulation, sports); practical exercise (audio): Decelerated breathing combined with mindfulness meditation; homework: continue pursuit of health goals, daily breathing exercises, regular blood pressure measurements; symbolic reward for progress (“puzzle piece of the day”).
**Recognizing and overcoming obstacles**	Identification of hurdles and obstacles in the context of a personally relevant example (e.g. difficulties in increasing physical activity due to lack of motivation, lack of time); discussion to the behavior change technique of mental contrasting (visualizing the goal, possible obstacles and options for overcoming the obstacles); clarification of theoretically relevant variables for the implementation of important health goals (expectations, subjective difficulty, social support); interactive clarification of goal progress and, optionally, personally relevant techniques and further information to support goal implementation (e.g. simple circuit training plan for patients who strive to become more physically active); audio exercise: mental imagery exercise on overcoming personal obstacles (combined with slow breathing and mindfulness meditation); homework: Continue pursuit of health goals, daily breathing exercises; symbolic reward for progress (“puzzle piece of the day”).
**Strengthening your impulse control**	Brief exploration of goal progress; case examples to illustrate the topic of “impulse control” (story of Claire / Philipp: “The daily struggle between the inner Dr. Jekyll and Mr. Hyde”); clarification of the personal relevance of the topic ("What does your inner Dr. Jekyll say these days?"); cognitive-behavioral technique for dealing with self-sabotaging or unhealthy thoughts and impulses: applying a “cognitive defusion” technique from Acceptance and Commitment Therapy, ACT); Audio exercise: coping with unhealthy impulses with cognitive defusion; Homework: continue pursuit of personal health goals; Worksheet: Identifying situational triggers of impulsive thoughts and using cognitive defusion; symbolic reward for progress (“puzzle piece of the day”).
**Development of an individualized day plan**	Brief exploration of goal progress and homework; interactive development of an ideal "personal day plan" with concrete instructions for health-promoting behavior (applying behavior change techniques of "action planning" and "implementation intentions"); e.g. individualized instructions for coping with sleep difficulties; implementing a balanced diet at different times of the day; scheduling times for exercise / physical activity and relaxation; planning time for blood pressure measurements; option to further customize the daily schedule by filling in free text fields; provision of the day-plan as a PDF document; audio exercise: mental imagery of an optimal day; homework: using your day-plan; symbolic reward for progress (“puzzle piece of the day”).
**Relapse prevention**	Interactive exploration of the personal meaning of "slips/setbacks" or "relapses" (e.g. resumption of unhealthy eating habits); normalization and validation of setbacks; early detection of potential relapses using a phase model (early signs of mental, emotional and behavioral relapse); coping with relapses through the technique of self-compassion; audio exercise “experiencing self-compassion”; interactive development of self-supporting statements (technique of “positive self-talk”); provision of an individual relapse prevention plan (also as a PDF document); symbolic reward for progress (“puzzle piece of the day”).
**Summary and conclusion**	Review of essential contents and techniques from previous modules in the context of a “fantasy journey” (application of the mental imagery technique “memory palace/method of loci”); motivating final audio: "Supporting your own blood pressure and health by maintaining health-promoting behavioral habits over the long term"; symbolic reward for progress (“final puzzle piece”).

Whereas participants randomized to the IG received access to *liebria* in addition to TAU, participants randomized to the CG only received TAU for the duration of the study, and were offered access to *liebria* after study completion.

TAU consisted of the regular care provided by the participant’s general practitioner and could, but did not have to, include antihypertensive medication or other treatment elements. Depending on the stage of hypertension and cardiovascular risk, general practitioners can recommend a therapy via initial education about hypertension and the importance of health-promoting behavioral habits. Moreover, with increased risk and/or blood pressure, a combination of behavioral changes with first-line antihypertensive medication can be advised or administered respectively. Patients of both groups were free to continue or discontinue any treatment elements, as they would in normal routine settings.

### Measures

#### Primary endpoint

The primary endpoint was SBP in mmHg measured via HBPM using a clinically validated, semi-automatic arm cuff device. Measurement of BP followed European clinical guideline criteria for home BP monitoring [45]. Patients were trained using an illustrated manual provided by trial staff. At each time point, participants were instructed to measure BP on three different (if possible, consecutive) days, twice per day (in the morning within one hour after awakening and in the evening at least two hours after dinner). For each measurement, three different BP readings were obtained, with a one-minute interval of rest in between measurements. Participants were instructed to rest for five minutes before starting the BP readings, to install the cuff at the upper arm (heart level) using the same arm as in previous office BP readings, to stick to that arm during the entire study, and to sit in a calm surrounding at room temperature with the back supported, arm resting on a table, without crossing legs, and if possible, without talking during the BP readings. Each result was entered manually by the patient to an online software. To exclude unstable results and lack of reproducibility due to uncertainties in handling and preparation [46], data from the first measurement day per time point and from the first reading per measurement on the remaining days were discarded. Remaining data (i.e., second and third reading in the morning and in the evening of day 2 and day 3; in total 8 readings) was averaged to yield BP per time point. The time point for the evaluation of effectiveness was after three months (T1).

#### Secondary endpoints

##### Responder rate

Responders were defined as participants achieving a reduction in SBP of at least 2.5 mmHg from baseline (T0) to T1, as agreed upon in personal communication with the German Federal Institute for Drugs and Medical Devices.

##### Diastolic blood pressure

Diastolic Blood Pressure (DBP; in mmHg) was obtained from the blood pressure readings described above.

##### Pulse pressure

Pulse Pressure (PP; in mmHg), which is related to the extent of atherosclerosis and considered to be a predictor of adverse cardiovascular outcomes [47], was calculated as SBP minus DBP.

##### Quality of life

Quality of life (QoL) was assessed using the WHOQOL-BREF [48]. The WHOQOL-BREF is a 26-item patient-reported outcome measure (PROM) that assesses quality of life in the past two weeks regarding four different domains (physical health, psychological health, social relationships, and environment). Items are answered on a Likert scale ranging from 1 (‘very dissatisfied’) to 5 (‘very satisfied’). After the scoring procedure, each domain score ranges from 0 (worst possible health state) to 100 (best possible health state).

##### Medication adherence

Medication adherence was assessed using the Rief Adherence Index (RAI), which is a four-item PROM addressing different aspects of medication adherence: throwing away medication, changing doses and discontinuation on one’s own account, discontinuation because of side effects [49, 50]. Items are answered on a Likert scale ranging from 5 (‘(almost) always happened (in 80–100% of cases)’ to 1 (‘(almost) never happened (in 0–20% of cases)’). The total score is obtained by adding single item scores and ranges from 4 (reflecting high adherence to prescribed medication) to 20 (reflecting high non-adherence).

##### Social and work-related functioning

Social and work-related functioning was assessed using the Work and Social Adjustment Scale (WSAS), a 5-item PROM measuring the ability to function in different contexts (work, home management, social leisure, private leisure, relationships) that presents good psychometric properties [51, 52]. Items are answered on a Likert scale ranging from 0 (‘not at all impaired’) to 8 (‘very severely impaired’), yielding total scores ranging from 0 (no impairment) to 40 (severe impairment).

#### Other measures

In addition to primary and secondary endpoints, sociodemographic and clinical variables were assessed via self-report. Acceptance and satisfaction with the treatment were evaluated using the net promoter score (NPS; [53]), which can range from −100 (very low satisfaction) to 100 (excellent satisfaction). The NPS was assessed only in the intervention group at the end of the study; positive NPS scores indicate good satisfaction.

### Sample size calculation

An a priori power analysis estimated that detecting a 4 mmHg difference in HBPM (SD = 16 mmHg), corresponding to an effect size of Cohen’s d = 0.25, was a realistic target [17, 20, 54], which would require 253 participants per group for 80% power at α = .05. To account for expected dropouts, the initial target sample was therefore set at *N* = 676 (*n* = 338 per group).

### Statistical analyses

All analyses were performed in R, version 4.2.1 [55]. Missing data were replaced using bootstrapped maximum likelihood multiple imputation [56] under the assumption of ‘missing at random’ (MAR) using the respective variable values at baseline, group membership, and other sociodemographic and clinical variables (age, sex, intake of antihypertensive medication at baseline, psychotherapy status at baseline) as predictors. Imputations were conducted using the R package *mice* [57].

Outcomes were analyzed according to the intention-to-treat (ITT) principle, including all randomized participants with imputation of missing data. Effectiveness was analyzed using analysis of covariance (ANCOVA), adjusting for baseline values of the respective outcomes. Between-group effects are reported on the original scale and as standardized mean differences (Cohen’s *d*) to facilitate effect size comparisons. Responder rates were compared using χ^2^-tests. Effectiveness analyses followed a pre-specified statistical analysis plan developed before data analysis to ensure methodological rigor. However, subgroup analyses originally planned for the larger target sample were not conducted due to limited power. A complete-case analysis was also conducted to examine the robustness of the findings.

## Results

### Description of trial participants

Participants were recruited between October 2021 and May 2022 (follow-up period up to August 2022) via a disease-specific campaign orchestrated by a health insurance company (DAK-Gesundheit) in Germany. 102 subjects met inclusion criteria (mean age 54.5 years, 47.1% male) and were randomized and included in the final analysis. Due to problems with the recruitment campaign, the originally planned sample size could not be attained (for a detailed explanation please refer to the Discussion section). Participant characteristics are presented in Table 2. There was no statistically significant between-group difference in included variables.

**Table 2:** Participant demographics and clinical characteristics at baseline

	**control**	***liebria***	**total**	***p***
	*n* = 50	*n* = 52	*n* = 102	
**Sex (% male)**	52.0	42.3	47.1	.434
**Age**	54.1 (9.1)	54.9 (6.9)	54.5 (8.0)	.633
**Systolic blood pressure**	144.1 (8.5)	144.0 (7.5)	144.1 (8.0)	.957
**Diastolic blood pressure**	92.6 (4.8)	93.6 (4.9)	93.2 (4.9)	.298
**Pulse pressure**	51.5 (6.8)	50.4 (6.9)	50.9 (6.8)	.422
**BMI**	27.1 (4.9)	28.6 (4.7)	27.8 (4.8)	.117
**Antihypertensive medication intensity level (%)**				
None	40.0	40.4	40.2	1
Monotherapy	42.0	38.5	40.2	.871
Dual combination therapy	12.0	17.3	14.7	.633
Triple combination therapy	6.0	3.8	4.9	.964
**Type of antihypertensive medication** ^**a**^ ** (%, multiple answers possible)**				
Renine-angiotensine- Aldosterone system blocker	52.0	48.1	50.0	.843
Antihypertensives^b^	6.0	2.0	4.0	.582
Beta blocking agents	8.0	11.5	9.8	.789
Calcium channel blockers	16.0	11.5	13.7	.714
Diuretics	2.0	4.0	2.9	1
**Psychotherapy (% yes)**				
Current	10.0	6.0†	8.0	.712
Ever	16.0	12.0^c^	14.0	.773

Note: Values represent mean (SD) unless stated otherwise.

^a^ classified based on the Anatomical Therapeutic Chemical (ATC) Classification System.

^b^ ATC class C02.

^c^ based on *n* = 50 due to missing data

### Intervention delivery and drop-out

**Fig. 1:**
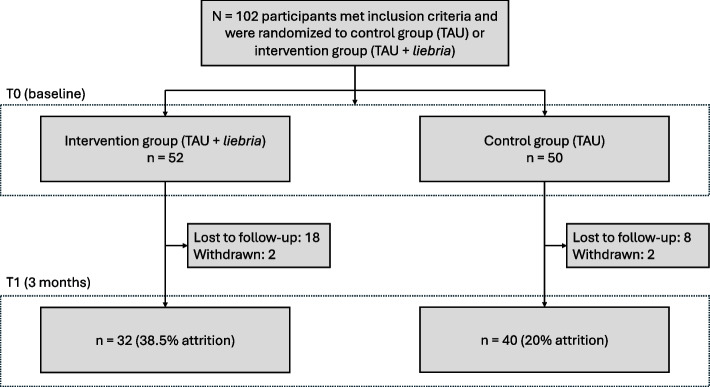
Study Flow Chart. Note: TAU = treatment as usual

*N* = 102 met inclusion criteria and were randomized to the IG (*n* = 52) or CG (*n* = 50). The drop-out rate at T1 was 29.4 % (38.5 % in the IG; 20 % in the CG). Reasons for drop-out are provided in the study flow chart (Fig 1). Of 52 participants randomized to the IG, 50 completed registration in *liebria* and used the intervention on at least one day*.*

### Study outcomes

#### Primary outcomes

The ITT analysis showed a treatment effect on SBP after 3 months of −3.5 mmHg (95% CI −7.1 to 0.13; *p* = .053; Cohen’s *d* = 0.36).

##### Secondary outcomes

Comparison of the number of responders (defined as a reduction in SBP of at least 2.5 mmHg from baseline to T1) in the ITT analysis revealed that clinically relevant effects on SBP were more frequent in the IG than in the CG (78.2% vs. 54.3%; *p* = .076). The observed difference in the reduction in SBP of at least 2.5 mmHg from baseline to T1 between intervention- and control group corresponds to a number needed to treat (NNT) of 5 (95% CI: 2.3 to 14.0).

The ITT analysis of the WSAS revealed treatment effects of liebria on social and work-related functioning after 3 months with a treatment effect of −1.3 (95% CI −2.3 to −0.3; *p* = .009; d = 0.40). No effects could be observed for DBP, PP, QoL, and medication adherence. See Table 3 for results of ITT analyses for all endpoints.

**Table 3:** Results of primary and secondary endpoints for ITT analyses

	**Time**	**control**			***liebria***			**ANCOVA**		
		n	mean	SD	n	mean	SD	Treatment effect (95% CI)^a^	*p*	Cohen’s *d* (95% CI)^b^
SBP (mmHg)	T0	50	144.1	8.3	52	144.0	7.4	−3.5 (−7.1; 0.13)	.053	0.36 (−0.06; 0.78)
	T1	50	140.3	9.6	52	136.7	10.7			
DBP (mmHg)	T0	50	92.6	5.1	52	93.7	4.8	−2.0 (−4.8; 0.8)	.166	0.16 (−0.30; 0.63)
	T1	50	90.1	6.8	52	89.0	9.5			
PP (mmHg)	T0	50	51.5	6.7	52	50.3	6.8	−1.9 (−4.5; 0.7)	.144	0.37 (−0.05; 0.79)
	T1	50	50.1	7.0	52	47.4	7.8			
WHOQOL-BREF Phys	T0	50	73.4	15.2	52	74.9	16.9	3.5 (−1.1; 8.2)	.138	0.27 (−0.12; 0.67)
	T1	50	72.4	19.6	52	77.1	15.1			
WHOQOL-BREF Psy	T0	50	67.5	16.3	52	69.3	15.7	1.6 (−4.0; 7.1)	.582	0.18 (−0.23; 0.59)
	T1	50	68.2	18.5	52	71.2	15.0			
WHOQOL-BREF Soc	T0	50	61.8	19.8	52	63.7	21.5	1.8 (−4.4; 8.8)	.565	0.16 (−0.24; 0.55)
	T1	50	62.4	22.5	52	65.7	20.1			
WHOQOL-BREF Env	T0	50	78.0	13.5	52	79.3	12.3	0.9 (−2.4; 4.2)	.604	0.15 (−0.26; 0.56)
	T1	50	79.1	13.6	52	80.9	9.8			
RAI	T0	50	6.2	2.9	52	5.0	1.8	−0.4 (−0.9; 0.2)	.198	0.49 (0.12; 0.86)
	T1	50	5.6	2.4	52	4.7	1.3			
WSAS	T0	50	4.5	4.9	52	4.2	5.8	−1.3 (−2.3; −0.3)	.009	0.40 (0.002; 0.81)
	T1	50	3.5	4.1	52	2.0	3.5			

*SBP* systolic blood pressure, *mmHg* millimeter mercury, *DBP* diastolic blood pressure, *PP* pulse pressure, *WHOQOL-BREF phys* World Health Organization quality of life measure, physical domain, *WHOQOL-BREF psy* World Health Organization quality of life measure, psychological domain, *WHOQOL-BREF soc* World Health Organization quality of life measure, social domain, *WHOQOL-BREF env* World Health Organization quality of life measure, environmental domain, *RAI* Reif Adherence Index, *WSAS* Work and Social Adjustment Scale

^a^group difference on the original scale three months after baseline, adjusted for baseline scores

^b^positive values show effects in favor of the intervention group

#### Adverse effects and user satisfaction

No adverse events or device effects were observed. 15.6% of participants in the IG compared to 37.5% of participants in the CG had a higher SBP at T1 compared to T0. The NPS was 7.9, indicating overall good satisfaction. Specifically, 73.3% of participants indicated good to very good satisfaction (7 or higher on the numerical rating scale ranging from 0 to 10).

## Discussion

The present study investigated the effectiveness of using the DTx *liebria* as an addition to TAU in reducing SBP and improving other relevant endpoints in adults with hypertension using a randomized design. The key finding is that participants who were offered *liebria* in addition to their usual care reported 3.5 mmHg lower SBP after three months, on average, compared to those who only received heterogeneous usual care, which is a clinically relevant effect comparable to established interventions. Although these results suggest a meaningful clinical signal, the small sample size poses a significant limitation.

The trial’s planned recruitment target of 676 participants was not achieved due to challenges in enrollment, which reduced the statistical power to detect significant effects for the primary outcome (SBP) and certain secondary endpoints. The observed reduction in SBP of −3.5 mmHg is consistent with effect sizes reported in similar intervention trials and suggests a clinically relevant outcome. However, the p-value for the primary outcome narrowly missed statistical significance (*p* = 0.053), likely due to the reduced sample size. This limitation is a key consideration for interpreting the findings and underscores the importance of adequate power in future studies.

Given the relatively small sample size and multiple comparisons, there is also an inherent risk of Type I error, meaning that some statistically significant findings may have occurred by chance. While the observed effects are in line with previous research, replication in larger, adequately powered trials will be necessary to confirm their robustness and clinical significance.

Enrolled subjects had mild hypertension at baseline, with mean SBP/DBP of 144/93 mmHg on home-based measurements. A significant proportion of the study population (40%) was not receiving any pharmacological treatment. As *liebria* uses psychological methods for blood pressure control, it was important to record the rate of current or previous psychotherapy for our study population, as this could have a biasing effect on the primary outcome. The majority of participants were naive to psychotherapy. As expected for a randomized design, there were no significant differences in baseline characteristics between groups.

In terms of primary and secondary outcomes, we found greater decrease in SBP and greater responder rate in the IG compared to the CG. There was an intervention effect on social and work-related functioning, but no treatment effects were observed on DBP, PP, QoL, and medication adherence. No adverse effects were observed, and user satisfaction was good.

The magnitude of the intervention effects on SBP is comparable to effects found in other randomized controlled trials (RCTs) investigating DTx for hypertension [17, 20, 54]. To put the treatment effect in a clinical context, the reduction in SBP of 3.5 mmHg is clinically relevant and comparable with other non-drug antihypertensive strategies, for example salt reduction, which resulted in a mean decrease in office blood pressure of 4.1 mmHg in a meta-analysis of clinical trials [58]. Usually, blood pressure reductions observed through home-based monitoring are smaller than those measured in office settings. Therefore, the treatment effect of liebria in this trial might have appeared larger if office BP measurements had been used. However, it is important to note that higher office BP readings may, in part, reflect measurement bias rather than a true effect, which would complicate interpretation.

A likely reason for missing statistical significance regarding SBP both in the ANCOVA and in the responder analyses is the smaller-than-planned sample size. Likewise, lacking statistical significance for the intervention’s effects on PP, physical QoL, and medication adherence potentially could also be attributed to insufficient power, given that effect sizes (*d* = 0.37, *d* = 0.27, and *d* = 0.49, respectively) are in the expected ranges for digital interventions for a variety of different endpoints [39, 41, 44, 59, 60]. Specifically, effects that we observed for physical QoL are comparable in magnitude to effects of online interventions for other indications [39, 41]. For PP, effects were smaller than those observed for other digital lifestyle interventions [35, 61]. Regarding medication adherence, effects were comparable and even slightly larger than those of other digital interventions that are specifically targeted at medication adherence in a variety of conditions [62].

*liebria* had significant effects on social and work-related functioning (*d* = 0.40; *p* = .009). This effect is slightly higher than the effects of an in-person health education and exercise program for people with hypertension on social functioning [63], and slightly lower than effects of digital health interventions for other indications [41]. The effect on social and work-related functioning might be particularly related to behavioral changes (e.g., increased physical activity by the means of an individualized day plan) that participants accomplished over the course of using the intervention.

*liebria* had a small and statistically non-significant effect on DBP. This is in line with other research showing significant effects of digital interventions on SBP, but not DBP [54]. However, the small effect should not be ignored. Considering the fact that the study population had only mild hypertension and *liebria* achieved the effect without any adverse effect, even a small DBP decrease by 2 mmHg compared to the control group is relevant in terms of clinical treatment concept. As a comparison, use of the DASH diet lowered office-measured DBP in the range of 1.7–2.1 mmHg in normotensives and in the range of 2.5–3.6 mmHg in hypertensives as reported in a meta-analysis of clinical trials [64].

For psychological, social, and environmental QoL, no effects of *liebria* could be observed (all *d*’s < 0.20, all *p*’s > .56). For the latter two, this is in line with expectations, given that a digital health intervention targeted primarily at individual health behavior is unlikely to have effects on personal relationships, social support, financial resources, or perceived safety in daily life. Regarding psychological QoL, improvements would have been more plausible, given that *liebria* targets not only health behavior but also includes breathing exercises, mindfulness meditation exercises, and CBT techniques for the modification of unhelpful thoughts. In fact, however, there is some evidence that lifestyle interventions specifically affect physical, but not mental, health-related QoL in patients with cardiovascular risk factors [65–67]. Nevertheless, these weak QoL-related effects suggest the need for further research to examine the reasons why liebria did not impact psychological QoL more strongly or consistently, and to consider adding elements to fortify the intervention in this respect. Several DTx developed by our team have shown effects on QoL and other psychological outcomes (38–42, 60) in various indications, such as multiple sclerosis, epilepsy, depression, and breast cancer, suggesting that it is possible, in principle, to leverage DTx for this purpose. Given the complexity of this challenge, it would be speculative and premature at this stage to determine whether or how *liebria* should be revised to enhance its effects on secondary outcomes, including QoL.

Several limitations ought to be noted. A key limitation is that the original recruitment goal of 676 participants could not be attained. The recruitment campaign, conducted through a single German health insurance provider, was less effective than anticipated. As a result, the study’s statistical power was low, and the sample may not fully represent broader populations. The smaller-than-planned sample size not only reduced statistical power for detecting effects on SBP but also likely influenced the findings for secondary outcomes. Non-significant effects on DBP, PP, and QoL do not necessarily indicate the absence of an intervention effect but may reflect insufficient power to detect smaller but potentially meaningful changes. Future studies with larger sample sizes are needed to clarify the intervention’s impact on secondary outcomes. Such trials could employ diversified recruitment channels, such as partnerships with multiple health insurers or direct-to-consumer campaigns, to improve participant reach. Expanding recruitment strategies in future studies could also enhance generalizability to diverse demographic and healthcare contexts.

An additional reason for the recruitment campaign’s limited success was that many interested patients acquired through the campaign seemed to have problems operating the online tool in which they should enter their last BP reading, resulting in an automated exclusion from the trial. The method of collecting BP data was revised for upcoming studies. Due to budget constraints after finishing the campaign, the study had to be stopped. This lower than targeted participant number reduced statistical power and therefore our ability to detect effects of *liebria* on SBP.

An additional limitation concerns the high drop-out rate, particularly in the intervention group (38.5% in the IG vs. 20% in the CG). Several factors may have contributed to this attrition. Engaging with a behavioral change program requires considerable motivation, and less motivated participants may have disengaged. Others may have found the program less helpful or not aligned with their expectations. Conversely, some participants may have discontinued use after experiencing early improvements, a phenomenon known as the 'good enough effect,' which has been observed in various behavioral interventions, including psychotherapy [68].

Beyond the intervention itself, study design factors may have played a role. Control group participants were informed they would receive access to *liebria* after study completion, which may have incentivized them to remain in the study, whereas those in the intervention group lacked such an incentive.

A detailed analysis of user engagement patterns—such as module completion rates or interaction levels—could provide further insights into how *liebria* engages users and where refinements could enhance adherence. However, such an analysis is beyond the scope of this paper. Future research should investigate specific engagement patterns and consider strategies such as gamification or real-time feedback to sustain motivation and reduce attrition. Unfortunately, the precise reasons for dropout remain speculative, as no direct data were collected from participants who withdrew.

To address the problem of missing data resulting from relatively high attrition, we used multiple imputation, which can introduce bias if data are not missing at random. However, we conducted complete-case analyses as a robustness check (see **Table S1** in the supplement), yielding comparable effect sizes. Of note, attrition is a well-known problem in trials examining digital interventions [41, 43, 59], and behavioral interventions in general [69–71]. However, the scalability and cost-effectiveness of a DTx for hypertension must be considered as well [72], as such interventions might have a considerable public health impact even when drop-out rates are relatively high.

An additional shortcoming of the study is that participants were not blinded to their group assignment, which could have led to biases such as expectancy effects, social desirability effects, or variations in the self-administration of home-based blood pressure measurements. Expectancy effects—where intervention participants anticipate positive outcomes—may have contributed to greater adherence to lifestyle changes or reporting biases. Additionally, self-monitoring bias could have influenced measurement accuracy, as participants in the intervention group may have been more engaged in tracking their BP than those in the control group.

To address this limitation, future studies should consider incorporating objective BP measurements (e.g., ambulatory BP monitoring) to improve reliability and reduce potential biases related to self-reporting. Additionally, researchers could explore partial blinding strategies, such as sham digital interventions or alternative control conditions, to minimize expectancy effects while maintaining feasibility.

We also acknowledge that this study relied on self-reported home blood pressure monitoring (HBPM) as the primary endpoint measurement. Although HBPM is widely used in hypertension research and clinical practice, it may introduce biases due to variations in adherence, measurement technique, and device accuracy. The inclusion of clinic-based or ambulatory BP monitoring as a secondary measure in future trials would enhance the objectivity of BP measurement and mitigate potential bias from self-reporting.

A further limitation of this study is that we did not adjust for or report detailed baseline data on comorbidities, such as coronary artery disease, atrial fibrillation, and obesity. While randomization is intended to balance such factors across groups, residual differences may still exist and could influence engagement and response to digital interventions. However, in accordance with CONSORT guidelines, we did not perform statistical tests for baseline differences, as any differences in an RCT occur due to randomization rather than systematic bias. Future research should prioritize more detailed baseline reporting for transparency, cross-study comparisons, and to further explore the role of comorbidities in digital health interventions.

Finally, we acknowledge the limitation that *liebria* was developed, funded, and distributed by GAIA, and several authors are affiliated with the company, which could raise concerns of potential conflicts of interest. While this study followed rigorous methodological procedures, including a pre-specified statistical analysis plan and scientific leadership by principal investigators unaffiliated with GAIA (KJC and AR), independent replication studies by research teams entirely unaffiliated with GAIA are recommended to confirm the effectiveness of the intervention.

## Conclusions

This RCT provides promising initial evidence for *liebria’s* effects on SBP and social and work-related functioning. High user satisfaction and the absence of adverse events further indicate its feasibility as a digital adjunct to hypertension management. The trial’s pragmatic design, with few exclusion criteria, supports the generalizability of findings. Future studies should replicate these effects in larger, sufficiently powered trials. Given that statistical significance for SBP was narrowly missed despite treatment effects comparable to similar interventions, future investigations should employ larger sample sizes, longer follow-up periods, and participants with more severe hypertension, alongside state-of-the-art BP measurement techniques. In sum, while statistical significance was not reached for the primary outcome, the observed SBP reduction and functional improvements suggest that *liebria* may be a valuable complement to usual care, warranting further investigation in more robust trials.

## Supplementary Information

Additional file 1.

## Data Availability

The data that support the findings of this study are available from the corresponding author upon reasonable request.

## Abbreviations

BP: Blood pressure
CBT: Cognitive behavioral therapy
CG: Control group
CVD: Cardiovascular disease
DBP: Diastolic blood pressure
HBPM: Home blood pressure measurement
IG: Intervention group
ITT: Intention-to-treat
MAR: Missing at random
NNT: Number needed to treat
PP: Pulse pressure
PROM: Patient-reported outcome measure
QoL: Quality of life
RAI: Rief Adherence Index
RCT: Randomized controlled trial
SBP: Systolic blood pressure
T0: Baseline measurement
T1: 3 Months after randomization
TAU: Treatment as usual
