# Supplementary material for: Internet-based digital intervention to support the self-management of hypertension compared to usual care: results of the HALCYON randomized controlled trial
Source: BMC Cardiovasc Disord. 2025 Apr 4;25:256. doi: 10.1186/s12872-025-04698-5 (PMC11971814; doi:10.1186/s12872-025-04698-5)
Supplement: Supplementary file 1 — Additional file 1. [file 12872_2025_4698_MOESM1_ESM.docx]

**Supplementary material to:**

**Internet-Based digital intervention to support the self-management of hypertension compared to usual care: Results of the HALCYON randomized controlled trial**

Content:

Supplementary Table S1. Results of primary and secondary endpoints for complete case sensitivity analyses.

**Table S1 |** Results of primary and secondary endpoints for complete case sensitivity analyses.

|  | **Time** | **control** | | | ***liebria*** | | | **ANCOVA** | | |
| --- | --- | --- | --- | --- | --- | --- | --- | --- | --- | --- |
|  |  | n | mean | SD | n | mean | SD | Treatment effect  (95% CI)^a^ | *p* | Cohen’s *d* (95% CI)^b^ |
| SBP (mmHg) | T0 | 40 | 143.7 | 9.1 | 32 | 143.1 | 5.7 | -3.5 (-7.5; 0.5) | .086 | 0.38 (-0.09; 0.84) |
|  | T1 | 40 | 140.2 | 10.0 | 32 | 136.2 | 11.4 |  |  |  |
| DBP (mmHg) | T0 | 40 | 92.9 | 5.2 | 32 | 93.2 | 4.9 | -1.9 (-4.6; 0.8) | .167 | 0.22 (-0.25; 0.69) |
|  | T1 | 40 | 90.4 | 6.7 | 32 | 88.8 | 7.8 |  |  |  |
| PP (mmHg) | T0 | 40 | 50.8 | 6.7 | 32 | 49.9 | 6.8 | -1.7 (-4.5; 1.1) | .233 | 0.31 (-0.15; 0.78) |
|  | T1 | 40 | 49.8 | 7.0 | 32 | 47.4 | 8.4 |  |  |  |
| WHOQOL-BREF phys | T0 | 40 | 73.9 | 15.9 | 32 | 76.3 | 16.6 | 4.4 (-0.9; 9.6) | .102 | 0.35 (-0.09; 0.80) |
|  | T1 | 40 | 72.3 | 20.9 | 32 | 78.5 | 13.6 |  |  |  |
| WHOQOL-BREF psy | T0 | 40 | 67.2 | 15.6 | 32 | 69.4 | 15.5 | 2.6 (-3.0; 8.3) | .361 | 0.25 (-0.20; 0.69) |
|  | T1 | 40 | 67.5 | 19.1 | 32 | 71.6 | 14.3 |  |  |  |
| WHOQOL-BREF soc | T0 | 40 | 62.8 | 20.4 | 32 | 63.7 | 20.4 | 1.6 (-5.2; 8.4) | .637 | 0.09 (-0.35; 0.53) |
|  | T1 | 40 | 63.2 | 23.1 | 32 | 65.1 | 19.7 |  |  |  |
| WHOQOL-BREF env | T0 | 40 | 77.8 | 13.7 | 32 | 79.6 | 11.6 | 0.6 (-3.0; 4.2) | .737 | 0.18 (-0.26; 0.62) |
|  | T1 | 40 | 78.9 | 14.1 | 32 | 81.0 | 9.3 |  |  |  |
| RAI | T0 | 40 | 5.8 | 2.3 | 32 | 5.0 | 1.9 | -0.4 (-0.9; 0.1) | .264 | 0.44 (-0.01; 0.88) |
|  | T1 | 40 | 5.5 | 2.5 | 32 | 4.6 | 1.3 |  |  |  |
| WSAS | T0 | 40 | 5.0 | 5.2 | 32 | 4.3 | 5.6 | -1.5 (-2.7; -0.3) | .014 | 0.51 (0.06; 0.95) |
|  | T1 | 40 | 4.3 | 4.3 | 32 | 1.8 | 3.4 |  |  |  |

*Note:* SBP = systolic blood pressure; mmHg = millimeter mercury; DBP = diastolic blood pressure; PP = pulse pressure; WHOQOL-BREF phys = World Health Organization quality of life measure, physical domain; WHOQOL-BREF psy= World Health Organization quality of life measure, psychological domain; WHOQOL-BREF soc= World Health Organization quality of life measure, social domain; WHOQOL-BREF env= World Health Organization quality of life measure, environmental domain; RAI = Reif Adherence Index; WSAS = Work and Social Adjustment Scale. ^a^ group difference on the original scale three months after baseline, adjusted for baseline scores; ^b^ positive values show effects in favor of the intervention group.

**Figure S1 |** Screenshot of a page of *liebria*


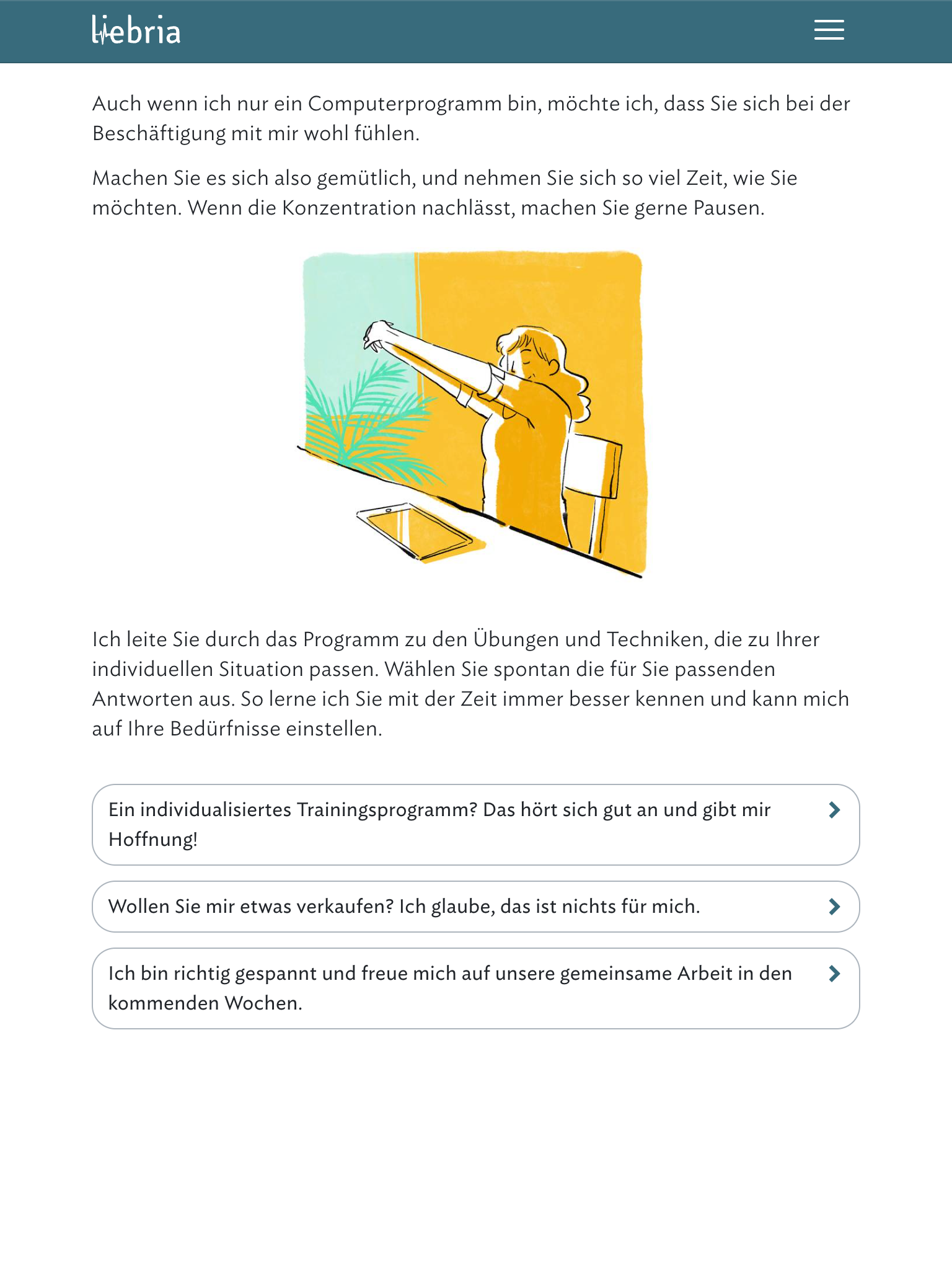


*Translation:*

1. Main text:

Even though I'm just a computer program, I want you to feel comfortable while engaging with me.

So make yourself cozy and take as much time as you need. If your concentration starts to fade, feel free to take breaks.

I’ll guide you through the program, introducing you to exercises and techniques that fit your individual situation. Choose the responses that feel right for you—this way, I’ll get to know you better over time and can adapt to your needs.

1. Response options:

- A personalized training program? That sounds promising and gives me hope!
- Are you trying to sell me something? I don’t think this is for me.
- I’m really excited and looking forward to working together over the coming weeks.
